# Extracellular Vesicles Derived from Adipose Mesenchymal Stem Cells Promote Peritoneal Healing by Activating MAPK-ERK1/2 and PI3K-Akt to Alleviate Postoperative Abdominal Adhesion

**DOI:** 10.1155/2022/1940761

**Published:** 2022-05-05

**Authors:** Manyu Shi, Hengchen Liu, Tingting Zhang, Mingzhao Zhang, Xin Tang, Zenan Zhang, Wenjun Lu, Shulong Yang, Zhitao Jiang, Qingbo Cui, Zhaozhu Li

**Affiliations:** Department of Pediatric Surgery, Second Affiliated Hospital of Harbin Medical University, No. 246, Xuefu Road, Nangang District, Harbin 150001, China

## Abstract

Peritoneal regeneration and repair can alleviate postoperative intraperitoneal adhesions, and mesenchymal stem cells (MSCs) have demonstrated the potential for peritoneal repair and regeneration. However, extracellular vesicles (EVs) are the main carriers for the MSC activity. Thus far, the roles of MSC-derived EVs on peritoneal repair are not well understood. To investigate the therapeutic effect of adipose-derived mesenchymal stem cell-derived EVs (ADSC-EVs) in peritoneal injuries, ADSC-EVs were injected in vivo via the tail vein of rats. The antiadhesion effects were evaluated following abdominal surgery. In addition, the levels of the peritoneal fibrinolysis system were determined via enzyme-linked immunosorbent assay. Expression differences in inflammatory and apoptotic markers were detected using immunofluorescence. The expression of extracellular matrix-related indexes and peritoneal healing were observed using immunohistochemistry. In vitro, rat peritoneal mesothelial cell proliferation was assessed via a 5-ethynyl-2-deoxyuridine assay. Cell migration was determined using scratch wound and transwell assays. Related signaling networks were estimated based on sequencing and bioinformatics analyses. The roles of the MAPK–ERK1/2 and PI3K–Akt signaling networks were analyzed using immunoblotting. This is the first report of the effectiveness of ADSC-EVs in the treatment of postoperative adhesions. ADSC-EVs were incorporated in vitro and induced rat peritoneal mesothelial cell proliferation and migration. This was mediated by stimulation of the MAPK–ERK1/2 and PI3K–Akt axes. ADSC-EVs promote the healing of the injured peritoneum, suggesting a promising therapeutic approach for peritoneal adhesions.

## 1. Introduction

Postoperative adhesions (PAs) are the most frequent and severe complications after almost any type of abdominal or pelvic surgery, as up to 90% of patients develop PAs after surgical procedures [1–3]. Severe PAs can cause serious complications like intestinal obstruction, persistent abdominal pain, and even female sterility [4, 5]. Peritoneal mesothelial cells are metabolically active and play an important role in maintaining serosal homeostasis. A recent report suggested that the mesothelial smooth surface protects against adhesion formation [6]. Peritoneal mesothelial repair differs from the repair of other epithelioid surfaces in that the repair is spread over the injured surface, whereas epithelial healing occurs only at the wound margin [7]. Additionally, the integrity of the mesothelial cells recovers relatively faster, regardless of the size of the wound. These observations suggest the existence of a unique healing mechanism [8], in which the cells not only migrate from the wound edge to the wound surface but also separate from opposite surfaces and distant locations to remain on the wound surface. The mitosis rate of the mesothelial cells at the wound edge increased from 0.16–0.5% to 28–60% at 24–48 h postinjury [9, 10]. Recent data have shown that the recovery of surgically injured mesothelial tissue reduces peritoneal adhesion and fibrosis, thereby improving the repair and function of the peritoneal structure [11, 12]. Therefore, promoting the recovery of the peritoneal mesothelial cells might serve as a new therapeutic strategy for reducing PAs.

Mesenchymal stem cells (MSCs) are a subset of adult stromal cells with multidirectional differentiation potential that have demonstrated great potential in preclinical studies [13, 14]. Adipose-derived mesenchymal stem cells (ADSCs) are abundant and convenient to obtain, which makes them more conducive for clinical applications [15, 16]. Moreover, ADSCs are commonly used in tissue repair and injury healing [17, 18]. Mounting evidence revealed that ADSC administration can significantly enhance the repair and regeneration of the peritoneum [19–22].

Recent studies have shown that the therapeutic effects of stem cells are mediated in part by extracellular vesicles [23]. Extracellular vesicles (EVs) are small membranous vesicles that contain a variety of proteins, DNA, mRNAs, and microRNAs (miRNAs) [24], which are important mediators of intercellular communication and play essential roles in immune regulation, cell migration, and tissue regeneration [25–27]. Recent findings indicate that the EVs released by MSCs modulate inflammatory responses [28, 29] and promote wound healing [30, 31]. However, the ability of MSC-derived extracellular vesicles (MSCs-EVs) in resolving peritoneal injury and postoperative adhesion is unclear.

We speculated that ADSC-EVs would relieve acute peritoneal injury by promoting peritoneal injury healing and extracellular microenvironment. To test this hypothesis, we examined the ADSC-EV-mediated regulation of (1) inflammation, regeneration, healing, the extracellular matrix (ECM), and PA healing after peritoneal injury in vivo as well as (2) the proliferation and migration of peritoneal mesothelial cells and the related mechanisms in vitro.

## 2. Materials and Methods

### 2.1. Animals

Male Sprague–Dawley (SD) rats were obtained from the Animal Experimental Center of Harbin Medical University (Harbin, Heilongjiang, China). All animal protocols strictly followed the guidelines of the Care and Use of Laboratory Animals of the United States National Institutes of Health, and our work received ethical approval from the appropriate committee (approval number Ky2018-135). Rats were randomly placed into one of three groups (six rats/group) and were euthanized at 4, 8, and 15 days postsurgery for the corresponding experiments. All rats were house4d in cages with constant humidity (60 ± 5%) and temperature (23 ± 2°C) with a 12 h light-dark cycle and free access to laboratory feed and water.

### 2.2. ADSC and Rat Peritoneal Mesothelial Cell Harvesting and Identification

ADSCs were extracted as reported previously [19]. Male SD rats, with body weights between 100 and 150 g, were euthanized via 2% sodium pentobarbital (40 mg/kg) intraperitoneal injection. Following 75% alcohol-mediated disinfection, the skin was incised diagonally at the groin to remove the subcutaneous fat. The adipose tissue was rinsed three times with sterile cold phosphate-buffered saline (PBS) and chopped. The ECM was lysed in 0.2% type-I collagenase (Sigma-Aldrich, St. Louis, MO, USA) for 1 h at 37°C, before centrifugation at 1000 × *g* for 10 min. ADSCs were cultured in DMEM/F12 (Invitrogen, Carlsbad, CA, USA) with 10% fetal bovine serum (FBS; Biological Industries, Kibbutz Beit-Haemek, Israel) and 1% penicillin-streptomycin (Beyotime, Shanghai, China) and incubated at 37°C and 5% CO2. Following a 48 h incubation, the medium was changed twice per week, with care taken to not remove the adherent cells. Phenotypic analysis was performed at passage 3 to begin generating ADSCs. Flow cytometry was employed to detect levels of different surface markers. The ADSCs were cultured separately in adipogenic, osteogenic, or chondrogenic differentiation media (Cyagen, Santa Clara, CA, USA) to identify their differentiation potentials.

Rat peritoneal mesothelial cells (RPMCs) were isolated as previously described [32]. Briefly, 25 mL of 0.25% trypsin and 0.02% EDTA-Na2 were injected into the intraperitoneal region of the rats. Peritoneal effusions were collected 2 h later under aseptic conditions. The isolated RPMCs were grown in DMEM/F12 medium with 20% FBS at 37°C and 5% CO_2_. RPMCs were verified based on certain characteristics, such as a polygonal pebble morphology and the presence of the mesothelium-specific markers cytokeratin-19, vimentin, and E-cadherin. Cells between passages 2 and 3, grown as a monolayer to 80% confluence, were employed in subsequent examinations.

### 2.3. Isolation, Identification, and Analysis of ADSC-EVs

Once the ADSCs achieved 80% confluency, the old culture medium was removed and Exo-depleted medium was introduced. After 24 h, the culture medium was collected without ADSCs before sequential centrifugation at 300 × *g* for 10 min, 3000 × *g* for 10 min, 10,000 × *g* for 30 min, and 100,000 × *g* for 2 h for Exo extraction. The EVs were mixed in PBS, and the total protein concentrations in the Exo samples were determined via a bicinchoninic acid assay (Beyotime). Nanoparticle tracking analysis, transmission electron microscopy (TEM), and western blotting were employed to characterize the isolated EVs. The aforementioned experiments were repeated three times.

### 2.4. Cellular Internalization of ADSC-EVs

ADSC-EVs were exposed to 1 *μ*M PKH26 (Sigma-Aldrich) for 5 min, and ultracentrifugation was performed to remove the excess dye. The labeled EVs were added to serum-free RPMC culture medium and maintained overnight in an incubator. Hoechst 33342 dye (UE, China, Suzhou) was used to stain the nuclei, and the images were subsequently captured with a fluorescence microscope (Leica, Wetzlar, Germany).

### 2.5. miRNA High-Throughput Sequence and Data Analyses

The miRNAs in ADSC-EVs were sequenced at OE Biotech Company (Shanghai, China). In short, ADSC-EV miRNA was isolated with the RNA isolation Kit (Takara), and the extraction quality was checked using an Agilent 2100 Bioanalyzer (Agilent Technologies). Next, 20 ng of Exo RNA was isolated for library construction. Finally, the libraries were sequenced with an Illumina HiSeq 2500 instrument (Illumina, San Diego, CA, USA). The top 10 miRNAs exhibiting the highest expression of ADSC-EVs were studied using miRanda software to predict target genes. DAVID and KOBAS 3.0 were employed for Gene Ontology (GO) and Kyoto Encyclopedia of Genes and Genomes (KEGG) pathway-enrichment analyses for the target genes.

### 2.6. Experimental Design and Surgical Procedures

All animals were operated upon as described previously [33]. The rats were fasted for 8 h before surgery. The male SD rats, with body weights ranging from 200 to 250 g, were anesthetized via intraperitoneal injection with 2% sodium pentobarbital (40 mg/kg). After disinfection with 75% alcohol, a median abdominal incision of approximately 2.0 cm was made on the skin. The cecum was extracted and rubbed gently with two pieces of dry gauze on the ventral and dorsal sides until luster was lost and bleeding spots were visible. The cecum was then replaced, and the layers were closed. Rats (*n* = 54) were arbitrarily separated into the following three groups: (1) sham—only the abdominal incision was performed, which was stratified and closed after 3 min of exposure; (2) ADSC-EVs—400 *μ*g ADSC-EVs were dissolved in 100 *μ*L PBS and administered via the tail vein for three consecutive days after abdominal closure; and (3) control—the same volume of PBS (100 *μ*L) was injected through the tail vein for three consecutive days after abdominal closure. After 4, 8, or 15 days, animals from each group (*n* = 6) were euthanized, and adhesion was evaluated in all animals using a U-shaped incision. After adhesion grading was performed, the tissues and peritoneal fluid were collected for subsequent experiments.

### 2.7. Peritoneal Adhesion Scoring System

After euthanasia at 8 and 15 days following peritoneal scraping, the degree of postoperative intraperitoneal adhesion was assessed using the adhesion scoring criteria proposed by Nair et al. [34]. Nair's score (see Table 1) was used to evaluate the degree of postoperative intraperitoneal adhesion. The grade evaluators were third-party contractors who were blinded to the research design.

### 2.8. Enzyme-Linked Immunosorbent Assays (ELISA) for Tissue Plasminogen Activator (t-PA) and Plasminogen Activator Inhibitor-1 (PAI-1) Levels in Peritoneal Fluid

Briefly, peritoneal fluids were collected from the abdominal cavities of the rats 8 days after peritoneal scraping. The contents of liquid-related factors, t-PA, and PAI-1 in the peritoneal fluid were determined using t-PA and PAI-1 ELISA kits (Shanghai Jianglai Biotechnology Inc., Shanghai, China), following the kit operational guidelines.

### 2.9. Histological Analysis of Peritoneal Adhesions

The rats were sacrificed at 4, 8, and 15 days after scraping (*n* = 6 at each time point/group). When fibrous bands did not form, the cecal wall was taken as the specimen; otherwise, the entire fibrous zone was taken. The specimens were immersed in formalin for 24 h, embedded in paraffin, and sliced into 4 *μ*m thick sections. For histology, all specimens underwent hematoxylin and eosin (H&E) staining using routine procedures. The observation was performed under a light microscope (Nikon Eclipse Ni-U, Tokyo, Japan) at 100x and 200x magnifications. Five randomly selected areas in each section were evaluated by an independent pathologist at 200x magnification.

### 2.10. Immunohistochemical Evaluation of Peritoneal Adhesions

For immunohistochemical analyses, the sections were exposed overnight at 4°C to antibodies against E-cadherin (rabbit monoclonal; 1 : 200; ab76319; Abcam), COL I (rabbit monoclonal; 1 : 200; ab270993; Abcam, Cambridge, UK), MMP-9 (rabbit monoclonal; 1 : 200; ab76003; Abcam), TIMP-1 (rabbit polyclonal; 1 : 200; Abcam), *α*-SMA (mouse monoclonal; 1 : 200; ab7817; Abcam), or fibronectin (rabbit monoclonal; 1 : 200; ab199056; Abcam), followed by incubation with secondary horseradish peroxidase-conjugated goat anti-rabbit IgG antibody (1 : 500; 115-035-003; Jackson ImmunoResearch, Ely, UK).

### 2.11. Immunofluorescence Staining of Peritoneal Adhesions

Next, we stained sections with immunofluorescence markers for inflammation and apoptosis as described previously [35]. In short, following a blocking step, the sections were exposed overnight at 4°C to antibodies against CD163 (rabbit monoclonal; 1 : 100; ab182422; Abcam), C-C chemokine receptor type 7 (CCR7; rabbit monoclonal; 1 : 200; ab32527; Abcam), interleukin- (IL-) 10 (rabbit monoclonal; 1 : 100; ab33471; Abcam), IL-6 (mouse monoclonal; 1 : 100; TA500067; Origene), COX-2 (rabbit monoclonal; 1 : 100; ab179800 Abcam), and cleaved caspase-3 (rabbit monoclonal; 1 : 100; ab32042; Abcam). Following a PBS-wash, the sections were exposed to a secondary antibody (1 : 200; SA00003 and SA00009; Proteintech) in the dark for 1 h, before being counterstained with 4,6-diamidino-2-phenylindole (DAPI, Beyotime) for 10 min. Image capture was performed with an Eclipse Ni-U microscope (Nikon), and analysis was carried out with ImageJ software.

### 2.12. Treating RPMCs with ADSC-EVs

To establish a peritoneal injury healing model in vitro, 1 × 10^6^ RPMCs were incubated in 6-well culture plates for 18 h, after which they were randomly divided into four groups. ADSC-EVs were added at concentrations of 0, 25, 50, or 100 *μ*g/mL after replacing the RPMC medium with an Exo-free medium. Next, to further investigate the associated mechanisms, RPMCs inoculated on a 6-well culture plate were arbitrarily separated into four groups as follows: (1) control group—the RPMC medium was replaced with Exo-free medium; (2) ADSC-EVs group—the RPMC medium was replaced with Exo-free medium containing 100 *μ*g/mL ADSC-EVs; (3) ADSC-EV+LY group—RPMCs were treated with 10 nM LY294002 (a PI3K/Akt inhibitor; MedChemExpress, Monmouth Junction, NJ, USA) for 30 min before adding 100 *μ*g/mL ADSC-EVs; and (4) ADSC-EV+PD group—RPMCs were treated with 50 nM of the MAPK/ERK1/2 inhibitor PD98059 (MedChemExpress) for 30 min before adding 100 *μ*g/mL ADSC-EVs. The RPMCs in each group were collected either 30 min or 24 h later and analyzed by western blotting. In addition, 5-ethynyl-2-deoxyuridine (EdU), scratch wound, and transwell assays were conducted 24 h later.

### 2.13. Proliferation Analysis of RPMCs

RPMC proliferation was assessed with the EdU Assay Kit (UE), following kit operational guidelines. Briefly, RPMCs (1 × 10^4^/group) were exposed to 50 *μ*M EdU for 1 h, fixed in 4% paraformaldehyde, and stained using the EdU kit. Hoechst dye (UE) was applied for 20 min to stain the cell nuclei. Finally, quantification of EdU-positive cells was performed manually using a fluorescence microscope.

### 2.14. Migration Analysis of RPMCs

To examine the cell migratory patterns, we selected scratch wound and transwell assays. In the scratch wound experiments, ADSCs (1.5 × 10^5^ cells/well) were plated in 6-well plates and allowed to attach overnight. A linear wound was generated in the monolayer using a sterile 200 *μ*L pipette tip. A serum-free medium containing ADSC-EVs was then introduced to each well. Image capture was performed at 0 and 24 h following ADSC-EV exposure with the help of an inverted microscope.

For the transwell experiments, RPMCs (1 × 10^4^/group) were plated in the upper chamber of the transwell plate (Corning Inc., NY, USA). Then, a medium with 10% Exo-free serum and different concentrations of ADSC-EVs was introduced into the lower chamber. Following 24 h, RPMCs on the permeable membrane of the upper chamber were subjected to fixation in paraformaldehyde and staining with crystal violet. Finally, the cells that traveled to the other side of the membrane were counted under a light microscope.

### 2.15. Western Blotting Analysis

ADSC lysis was performed in RIPA buffer (Beyotime) for protein extraction. The protein concentrations in the lysates were estimated by performing dipicolinic acid assays (Beyotime). Immunoblotting was performed with primary rabbit antibodies against TSG101 (monoclonal; 1 : 2000; ab125011; Abcam), CD9 (monoclonal, 1 : 2000; ab92726; Abcam), Hsp70 (monoclonal; 1 : 1000; ab2787; Abcam), ERK1/2 (1 : 1000; Cell Signaling Technology), phospho-ERK1/2 (1 : 2000; Cell Signaling Technology), Akt (1 : 1000; Cell Signaling Technology), and phospho-Akt (1 : 2000; Cell Signaling Technology). A horseradish peroxidase-conjugated goat anti-rabbit antibody (1 : 5000; Boster, China) was employed as the secondary antibody. ImageJ software was used for 4densitometric analysis of the final protein bands.

### 2.16. Statistical Analyses

All data are presented as the mean ± standard deviation. Quantitative data across all groups were compared using one-way analysis of variance and Tukey's test. *P* < 0.05 was set as the significance threshold.

## 3. Results and Discussion

### 3.1. Characterization of ADSCs, ADSC-EVs, and RPMCs

**Figure 1:**
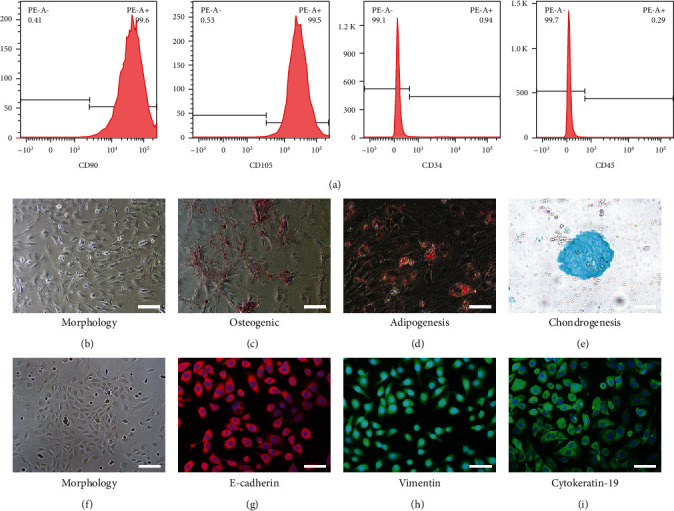
Characterization of ADSC and RPCM. (a) Flow cytometry for detection of ADSC surface markers, including CD90, CD105, CD34, and CD45. (b) Morphology of ADSC. (c) Osteogenic. (d) Adipogenic. (e) Chondrogenesis differentiation potential of ADSC. (f) The phenotype of rat peritoneal mesothelial cells (RPMCs) in culture. Immunofluorescence staining of RPMC. Mesothelial cells with (g) E-cadherin, (h) vimentin, and (i) cytokeratin-19. Original magnification: ×200.

**Figure 2:**
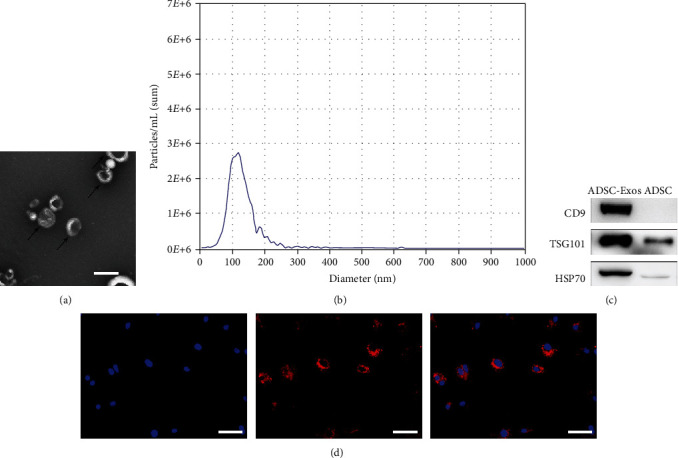
Characterization of ADSC-EVs. (a) Morphology was observed under a transmission electron microscope. (b) Particle size distribution. (c) Western blot was used to detect EVs surface markers. (d) fluorescent microscopy analysis of PKH26-labeled ADSC-EVs internalization RPMCs. Nuclei were counterstained with DAPI. Bars, 100 nm.

Flow cytometry was used to detect the positive markers CD90 (99.6%) and CD105 (99.5%), as well as the negative markers CD34 (0.94%) and CD45 (0.29%) (Figure 1(a)). ADSCs had a typical fusiform morphology and the potential for multidirectional differentiation (Figures 1(b)–1(e)). RPMCs had a typical cobblestone appearance (Figure 1(f)), and immunofluorescence staining results for E-cadherin, vimentin, and cytokeratin 19 were positive (Figure 1(g)–1(i)). TEM analysis revealed that ADSC-EVs had around, vesicle-like structure with a mean diameter of 120 nm (Figure 2(a) and 2(b)). Western blotting indicated the expression of CD9, TSG101, and HSP70 in EVs (Figure 2(c)). Moreover, ADSC-EVs were incorporated by RPMCs and displayed red fluorescence (Figure 2(d)).

### 3.2. Intravenous Injection of ADSC-EVs Reduces Adhesion Scores and Alleviates Inflammation in Acute Peritoneal Adhesions

**Figure 3:**
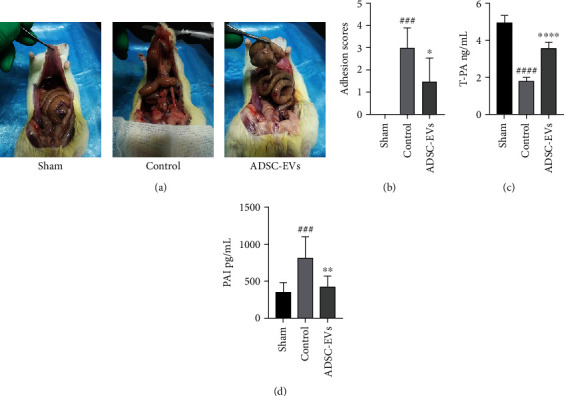
The Nair scores and fibrinolytic system for each group. (a) The severity of adhesions in each group. (b) The Nair score for each group (vs. control group, ^∗^*P* < 0.05; vs. sham group, ^###^*P* < 0.001). (c, d) t-PA and PAI-1 protein levels in peritoneal fluid. Data are expressed as the mean standard error of the mean (vs. ^∗∗^*P* < 0.01 and ^∗∗∗∗^*P* < 0.0001; vs. sham group ^###^*P* < 0.001 and^####^*P* < 0.0001).

Fifteen days after peritoneal scraping, abdominal adhesions were evaluated after laparotomy. The degree of adhesion differed significantly among the groups. Rats treated with PBS (control group) had dense adhesions and higher average adhesion scores. Adhesions were significantly reduced in rats injected intravenously with ADSC-EVs (Figure 3(a)). We observed marked differences in the scores and severity of adhesions between the groups (Figure 3(b)). However, at 8 days after peritoneal scraping, no obvious difference was detected between the treated and control groups. The results are not displayed.

### 3.3. ELISA Analysis of T-PA and PAI-1 Levels in 4Peritoneal Fluid

The expression of t-PA in the peritoneal fluid of the control group decreased significantly at 8 days after peritoneal scraping, and the expression of PAI-1 increased significantly. However, ADSC-EV treatment significantly increased t-PA expression and significantly inhibited PAI-1 expression (Figures 3(c) and 3(d)), relative to levels expressed in the control group.

### 3.4. Effects of ADSC-EV Treatment on Early Inflammatory Responses and Apoptosis

**Figure 4:**
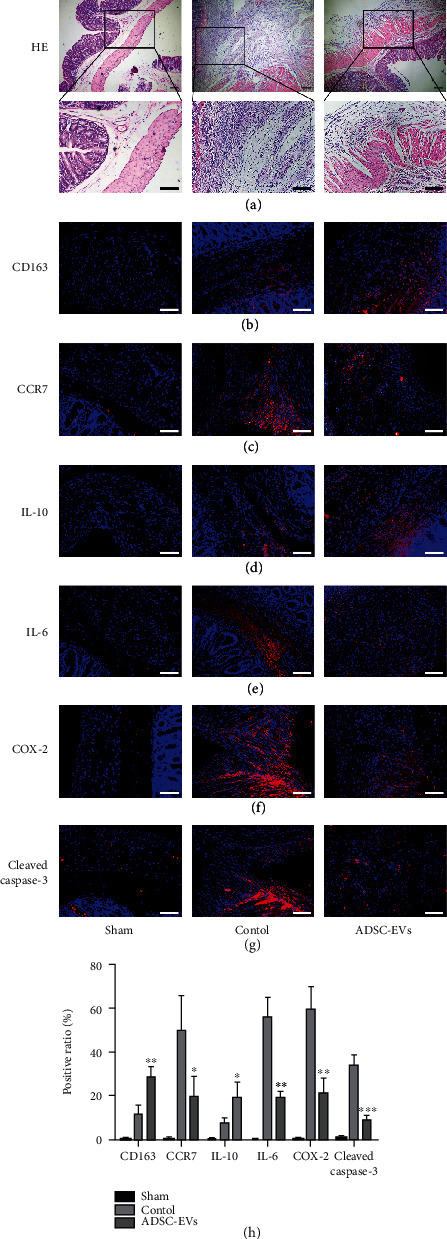
Impact of ADSC-EVs on peritoneal inflammation and apoptosis. (a) Hematoxylin-eosin staining of injured tissues (40x). Black tetragonum represents the accumulation of plasma cells, granulocytes, and macrophages (200x). (b–f) The expression of CD163^+^, CCR7^+^, IL-10^+^, IL-6^+^, and COX-2^+^ was evaluated at 4 days postsurgery by immunofluorescence assay. (g) Impact of ADSC-EVs on peritoneal apoptosis. Cellular expression of caspase 3^+^ at 4 days after surgery evaluated by immunofluorescence assay. (h) Positive cell ratio of inflammation-related factors and caspase 3+ cells at 4 days (*n* = 6). Bars, 50 *μ*m. Data are represented as mean ± SD. ^∗^, vs. control; ^∗^*P* < 0.05,  ^∗∗^*P* < 0.01, and^∗∗∗^*P* < 0.001.

We performed H&E staining to compare the degree of inflammation in the serosal layer on the cecal surface of rats in each group on day 8. The cecum serosal layer in the control group was significantly damaged, and plasma cells, granulocytes, and macrophages were significantly increased as observed by H&E staining (Figure 4(a)). Subsequently, we examined the significance of ADSC-EVs in regulating early inflammatory responses in rats. CD163 (an anti-inflammatory M2-type macrophage marker) expression was significantly elevated in the ADSC-EV-treated cells after 4 days, relative to that in controls (Figure 4(b)). In contrast, CCR7 (a proinflammatory M1-type macrophage marker) expression was significantly lower (Figure 4(c)). Correspondingly, IL-10 (an M2 macrophage-stimulating factor) levels in the ADSC-EV-treated cells were higher than those in controls (Figure 4(d)), whereas the expression of IL-6 (an M1 macrophage-stimulating factor) was significantly lower (Figure 4(e)). In addition, we found that Cox-2 (proinflammatory factor) expression was drastically lower in the treated cells (Figure 4(f)).

We then examined the effects of ADSC-EVs on the apoptosis profiles after peritoneal scratch injury. Based on our immunofluorescence data, cleaved caspase-3 (an apoptotic cell marker) levels in intestinal wall cells decreased significantly in the ADSC-EV-treated cells, relative to those in the injured controls (Figure 4(g)). A quantitative analysis of all of these results is shown in Figure 4(h).

### 3.5. ADSC-EVs Improve Peritoneal Healing and Modulate ECM Formation

**Figure 5:**
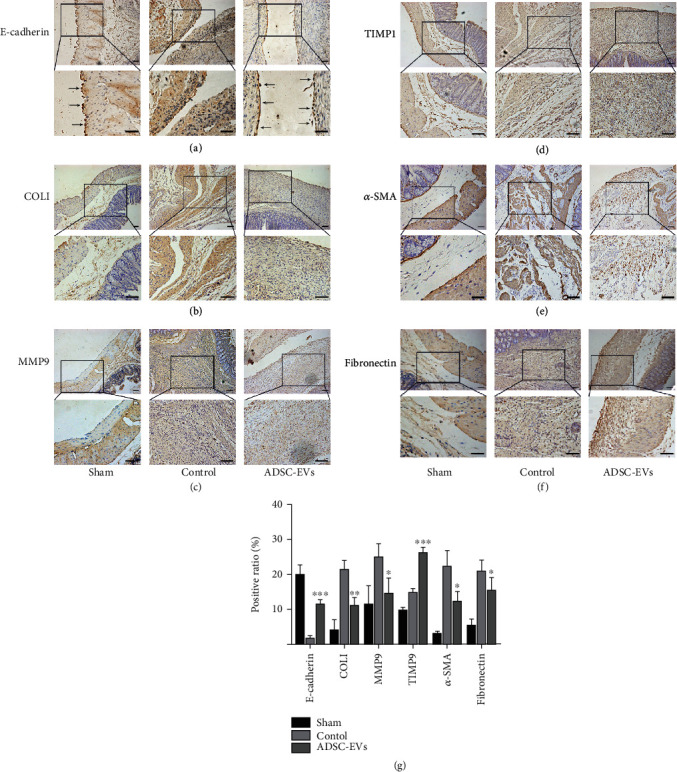
Impact of ADSC-EVs on peritoneal mesothelium cells and peritoneal matrix-related factors. (a) Immunohistochemical staining of E-cadherin in the injured areas peritoneum in each group of rats. RPMCs are shown by black arrow (100x in the upper plate, 200x in the below plate). (b–f) The expression of COL1, MMP-9, TIMP-1, *α*-SMA, and fibronectin was evaluated at 15 days postsurgery by immunohistochemistry assay (100x in the upper plate, 200x in the below plate). (g) Quantitative analysis of peritoneal matrix-related factors at 15 days. Bars, 50 *μ*m. Data are represented as the mean ± SD. ^∗^, vs. control; ^∗^*P* < 0.05,  ^∗∗^*P* < 0.01, and^∗∗∗^*P* < 0.0001.

To evaluate the regeneration ability of damaged peritoneal mesothelial cells in rats, we completed immunohistochemical staining for E-cadherin, a mesothelial cell marker, to evaluate the continuity of the peritoneal mesothelial layer in rats with cecal damage. In our study, a continuous and intact cell layer was detected on the caecum surface of the sham group. No mesothelial cells were observed on the injured peritoneal surface in the control group. Similarly, the mesothelial cell layer was well repaired in the ADSC-EV group, and its continuity was similar to that of the sham group. In the ADSC-EV group, repair of injured mesothelial cells on the peritoneal surface was significantly promoted (Figure 5(a)).

We also examined the significance of ADSC-EVs concerning the peritoneal ECM-related factors COL1, matrix metalloproteinase-9 (MMP-9), and tissue inhibitor of matrix metalloproteinase-1 (TIMP-1). On day 15, relative to control levels, TIMP-1 expression was elevated in the ADSC-EV treatment group (Figure 5(d)), while COL1 and MMP-9 expressions were downregulated (Figures 5(b) and 5(c)). The myofibroblast marker *α*-SMA showed a decreasing trend in the ADSC-EV treatment group at 15 days (Figure 5(e)). The presence of fibronectin was associated with scar formation after peritoneal injury, and fibronectin expression decreased drastically in the ADSC-EV-treated cells (Figure 5(f)). Quantitative analysis of these results is shown in Figure 5(g).

### 3.6. Cell Proliferation and Migration

**Figure 6:**
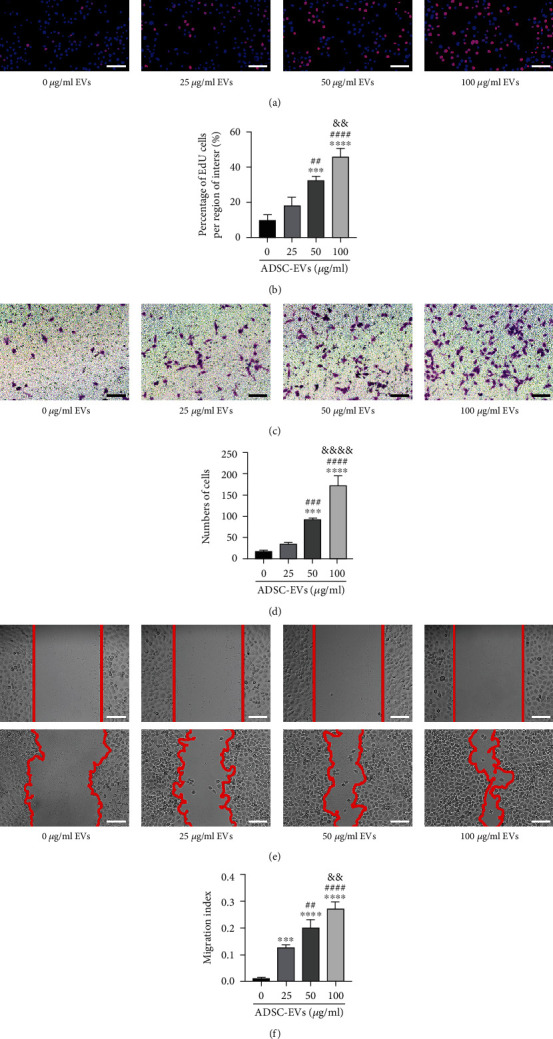
ADSC-EVs significantly promote the proliferation and migration of RPMCs in a dose-dependent manner. (a, b) Effect of different concentrations of ADSC-EVs on the proliferation of cells by EdU assays. The migration ability of RPMCs treated with ADSC-EVs, measured by (c, d) transwell and (e, f) scratch test assays. Bars, 100 *μ*m. Data are represented as the mean ± SD. ^∗^, vs. control group; *n* = 3. ^∗∗∗^*P* < 0.001. ^#^, vs. 25 *μ*g/mL group; ^##^*P* < 0.01 and ^##^*P* < 0.0001. ^&^, vs. 50 *μ*g/mL group; ^&&^*P* < 0.01 and ^&&&&^*P* < 0.0001.

Next, we assessed the outcomes of the two pathway inhibitors concerning RPMC proliferation and migration using different concentrations of ADSC-EVs. The results of EdU assays revealed that EVs remarkably accelerated cell proliferation in proportion with the dose (Figure 6(a)).

The transwell assay data showed that peritoneal mesothelial cell migration gradually increased 24 h following treatment with varying concentrations of EVs (Figure 6(c)). Scratch tests showed similar results (Figure 6(e)). A quantitative analysis of these results is shown in Figures 6(b), 6(d), and 6(f).

### 3.7. miRNA-Sequencing and Bioinformatics Analyses of ADSC-EVs

**Figure 7:**
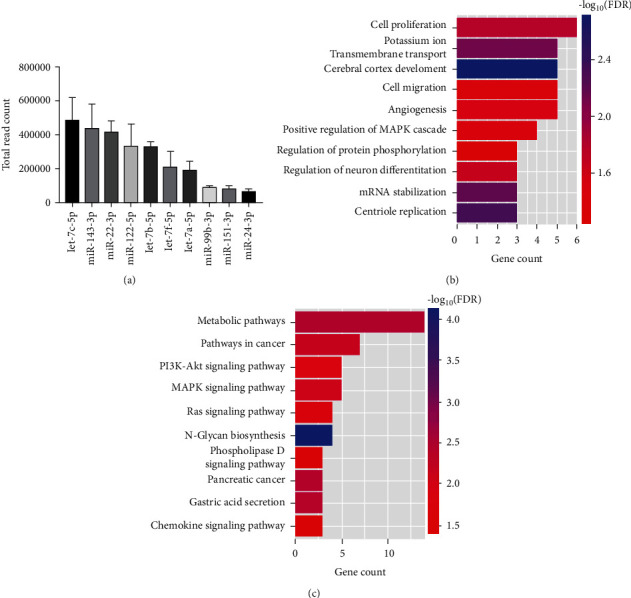
miRNA sequencing and bioinformatics analysis of ADSC-EVs. (a) The top 10 miRNAs that were detected in ADSC-EVs (*n* = 3). (b, c) GO and KEGG pathway enrichment analyses of the possible target genes.

To understand whether ADSC-EVs could promote RPMC proliferation and migration, we sequenced miRNAs in ADSC-EVs. We selected the top 10 miRNAs (Figure 7(a)) for functional-enrichment analysis using the GO and KEGG databases. The most enriched biological process (BP) categories were regulation of “cell proliferation” and “cell migration” (Figure 7(b)). KEGG analyses revealed that “PI3K/Akt” and “MAPK” were the most significantly enriched signaling pathways (Figure 7(c)).

Hence, we speculated that ADSC-EVs could accelerate RPMC proliferation and migration via the MAPK–ERK1/2 and PI3K–Akt signaling pathways. Schematic representation of predicted target genes and corresponding cellular functions of the miRNAs (Supplementary Fig. 1A), the most enriched molecular function (MF) category was “ATP binding” (Supplementary Fig. 1B), and the most common cellular component (CC) category was “membrane” (Supplementary Fig. 1C).

### 3.8. The Mechanism through Which ADSC-EVs Regulate RPMC Proliferation and Migration

**Figure 8:**
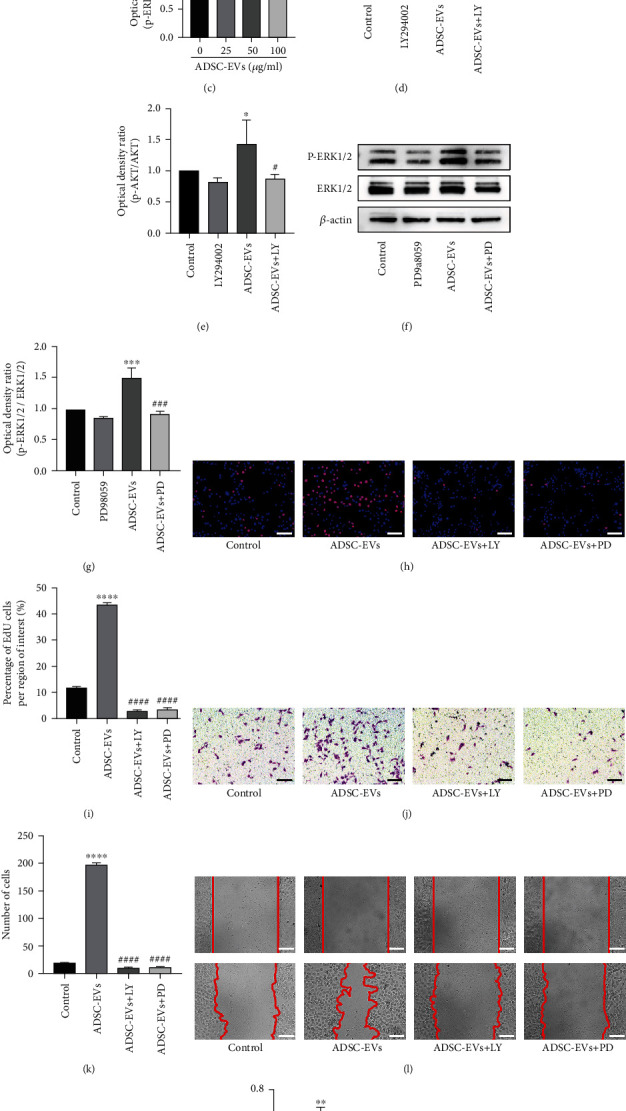
ADSC-EVs promote the proliferation and migration of cells via the PI3K/AKT and MAPK/ERK1/2 signaling. (a–c) Western-blot analysis of protein levels of p-Akt and p-ERK1/2 induced by different concentrations of ADSC-EVs. (d–g) LY294002 and PD98059 inhibit the activation of PI3K-AKT and MAPK-ERK1/2 induced by ADSC-EVs, respectively. (h, i) EdU assay showed that ADSC-EV-mediated RPMC proliferation was suppressed by inhibitors LY294002 and PD98059. (j, k) Transwell and (l, m) scratch assays showed that ADSC-EVs enhanced RPMC migration at 24 h, but this effect was significantly reduced by inhibitors LY294002 and PD98059. Bars, 100 *μ*m; data are represented as the mean ± SD. ^∗^, vs. control group; *n* = 3. ^∗∗∗^*P* < 0.001. ^#^, vs. 25 *μ*g/mL group; ^##^*P* < 0.01 and ^##^*P* < 0.0001. ^&^, vs. 50 *μ*g/mL group; ^&&^*P* < 0.01; ^&&&&^*P* < 0.0001.

Based on the sequencing results obtained with ADSC-EVs, we studied the MAPK–ERK1/2 and PI3K–Akt cell pathways to explore the mechanism whereby ADSC-EVs regulate the proliferation and migration of RPMCs. Western blotting analysis was performed to assess the activation of these two pathways. Based on our analysis, ADSC-EVs markedly increased p-ERK1/2 and p-Akt protein levels in RPMCs in a dose-proportionate manner (Figure 8(a)). These findings suggest that ADSC-EV-dependent proliferation and migration could be mediated by MAPK–ERK1/2 and PI3K–Akt signaling. To further investigate the regulatory effects of these two networks on RPMCs, we pretreated cells with the signaling inhibitors LY294002 and PD98059 before ADSC-EV exposure. In functional terms, the results of the EdU experiments showed that ADSC-EVs could significantly dampen the inhibitory effects of MAPK–ERK1/2 and PI3K–Akt signaling on RPMC proliferation (Figure 8(h)). Similarly, ADSC-EVs promoted peritoneal mesothelial cell migration, which was also markedly inhibited by both signaling inhibitors (Figures 8(g) and 8(l)). Based on our data, these signaling inhibitors significantly inhibited the ADSC-EV-induced phosphorylation of both Akt (Figure 8(d)) and ERK1/2 (Figure 8(f)).

At present, the main pharmacological measures used to prevent abdominal adhesions include (1) promoting fibrinolysis, (2) inhibiting fibroblast proliferation, (3) reducing the initial inflammatory response and exudation, (4) using a physical barrier to isolate the fibrin coating on the damaged surface, and (5) suppressing exudate agglutination. However, other investigators have reported that these treatments are not significantly effective [36]. The reason why these approaches are not effective in preventing adhesion might be that the functions of mesothelial cells on the peritoneal surface were neglected.

Mesothelial cells serve as a mechanical defensive barrier by covering the abdominal wall and internal organ surfaces with a single layer of flat epithelial cells to prevent subcutaneous tissue exposure and microbial invasion. During the early stage of abdominal adhesions, if effective procedures are adopted to improve the regeneration and migration capability of mesothelial cells such that a complete mesothelial layer is reconstructed, the formation of mature adhesions can be efficiently suppressed [11, 37, 38]. Moreover, mesothelial cells, which are essential proteases in the fibrinolytic pathway, are the main source of t-PA [39]. Therefore, increasing the number of mesothelial cells and the level of t-PA is one method of preventing adhesion. Mounting evidence suggests that MSCs can suppress the early inflammatory response following peritoneal injury [30] and accelerate mesothelial cell healing [40, 41]. Paracrine regulation is the main mechanism through which MSCs modulate peritoneal injury and mesothelial cell healing [21, 42, 43]. Continued research has identified EVs as the primary material mediating communication between cells. However, it is unclear whether MSC-EVs can regulate peritoneal injury and mesothelial cell healing. Our work in this study was the first to demonstrate that the intravenous administration of ADSC-EVs can promote peritoneal repair after acute peritoneal injury in rats; the results suggest that ADSC-EVs can promote peritoneal repair, which might provide a novel grounds for further exploration to treat and prevent PAs.

We first examined the role of ADSC-EVs in peritoneal injury and peritoneal adhesion, and we established a complete rat model of acute peritoneal adhesion. We revealed that ADSC-EVs significantly promoted peritoneal healing and prevented the formation of peritoneal adhesions in rats, relative to that in PBS-controls. In addition, with the rapid healing of peritoneal mesothelial cells, peritoneal fluid secretion of t-PA increased and PAI-1 secretion decreased, ultimately alleviating PAs. Taken together, this evidence indicates that the rapid enhancement of mesothelial cell regeneration is a potential approach for preventing adhesion formation.

The early inflammatory response has a crucial function in regulating peritoneal mesothelium healing. Macrophages are the most common cell type is damaged peritoneum tissue, and previous data showed that they are present on day 1 following adhesion induction [44]. Macrophage functions are primarily dependent on the differentiation state and can affect wound healing in multiple organs [45]. M1 macrophages play important roles in ECM degradation during the inflammatory response [46]. However, M2 macrophages participate in wound healing and tissue remodeling via ECM production [47]. In this study, 4 days after peritoneal injury, we found significantly fewer CCR7+ M1 macrophages and significantly more CD163+ M2 cells in the ADSC-EV-treated group after peritoneal injury in comparison to the corresponding levels in the other groups. In addition, our results revealed increased levels of the M2-stimulating factor IL-10, elevated inflammatory cytokine expression, and reduced IL-6 expression. Based on these results, we suggest that ADSC-EVs can promote anti-inflammatory activity and peritoneal injury healing by regulating the differentiation status of macrophages and related cytokines. Next, COX-2 (proinflammatory factor) has been strongly associated with adhesion and fibrosis after peritoneal injury [48]. In this study, COX-2 expression was significantly decreased in the treated rats after peritoneal injury, suggesting that ADSC-EVs are crucial to the repair and anti-inflammatory effects following peritoneal injury.

H&E staining showed that fewer neutrophils infiltrated the serosal tissue of the colon in the ADSC-EVs group. This finding indicated that ADSC-EVs positively affected the repair of peritoneal injury, which was further confirmed by the changes observed in ECM-related factors during the peritoneal repair. Peritoneal adhesions are caused by peritoneal injury, infection, ischemia, or oxidative stress. These stimuli can cause peritoneal mesoderm cell dysfunction, resulting in inflammatory exudation rich in fibrinogen, which is then degraded primarily by fibrinolysis. Adhesions form when fibrinolysis of the damaged peritoneum is insufficient owing to the excessive deposition of ECM components. Upregulation of fibrinolytic activity leads to decreased protein expression of the ECM, in which type-I collagen is the most important component [49]. The injury-control group showed much higher expression of type-I collagen than the sham-operation group, indicating that excess ECM proteins were deposited but that they could be reduced by administering ADSC-EVs. MMPs comprise a family of proteases for which their main activity is the degradation of the ECM. MMP-3, an important member of the MMP family, can inhibit PAI-1 expression and elevate plasminogen levels. In turn, plasminase activates ProMMP-9 to form MMP-9, which assists in remodeling the ECM [50]. TIMPs are natural inhibitors of the MMP family members, which inhibit MMPs by degrading the ECM. The balanced expression between MMPs and TIMPs has been reported to play an essential role in ECM remodeling [51, 52]. Here, we demonstrated that TIMP-1 expression was upregulated, whereas MMP-9 expression was downregulated, at 15 days after peritoneal injury in the ADSC-EV-treated group. Based on our data, ADSC-EV exposure mediates peritoneal ECM degradation and synthesis by regulating the metabolic balance between TIMP-1 and MMP-9. In addition, the levels of *α*-SMA and fibronectin were also detected, and they displayed a decreasing trend. Previous evidence showed that the overexpression of *α*-SMA and fibronectin inhibitors prevent postoperative scarring and fibrosis development after PA [53, 54]. In conclusion, our findings indicate that ADSC-EVs critically suppress the development of peritoneal adhesions and accelerate the rapid healing of mesenchymal cells after peritoneal injury.

In general, intravenous injection of EVs is the most common method of EVs delivery [55–57]. For example, the delivery of EVs prepared from mice human umbilical cord MSC-EVs (HUC-MSC-EVs) carrying miRNA-326 inhibits neddylation to relieve inflammatory bowel disease (IBD) [56]. In addition, Mao et al. [57] treated IBD in mice by injecting 400 *μ*g of (HUC-MSC-EVs) into the tail vein, and the results showed that extracellular vesicles could reach colon tissues after intravenous administration. Furthermore, intraperitoneal injection of the EVs has not been well studied; thus, we chose intravenous injection to transplant ADSC-EVs. In this study, we demonstrate that the systemic administration of ADSC-EVs can be used as a new treatment method for PAs. EVs have negligible immunogenicity in comparison to stem cell injection, and EVs have more advantages than stem cells.

We examined the significance of ADSC-EVs on the proliferation and migration of peritoneal mesothelial cells in vitro. We revealed that ADSC-EVs promote the proliferation and migration of peritoneal mesothelial cells in a dose-proportionate manner. MSC-EVs play a communication role by carrying various “messages” from parent cells to recipient cells, these “messages” include DNA, miRNAs, non4coding RNAs, proteins, and lipids [58]. EVs mostly function using miRNAs [59, 60] and proteins [61]. We sequenced miRNAs in ADSC-EVs to elucidate the mechanism whereby ADSC-EVs promote the proliferation and migration of peritoneal mesothelial cells. The top 10 most highly expressed miRNA target genes were predicted and analyzed using bioinformatics. Enrichment analysis revealed that “PI3K–Akt” and “MAPK” were the most enriched networks. The most enriched BP categories were regulation of “cell proliferation” and “cell migration.” Hence, we hypothesized that ADSC-EVs accelerate the proliferation and migration of peritoneal mesothelial cells by activating the MAPK–ERK1/2 and PI3K–Akt pathways. Consistent with this hypothesis, ADSC-EVs induced the rapid phosphorylation of Akt and ERK, which was abrogated in the presence of inhibitors. These data suggest that the prosurvival signals operating in RPMCs can be quickly stimulated by ADSC-EVs, thus improving their survival ability. We also revealed that ADSC-EV-driven proliferation and migration were downregulated by MAPK–ERK1/2 and PI3K–Akt pathway inhibitors. In summary, these data suggest the possibility that ADSC-EVs promote RPMC proliferation and migration by triggering activation of the MAPK–ERK1/2 and PI3K–Akt axes. However, our study only focuses on miRNA, and other components in EVs, such as proteins, lipids, or nucleic acids, may also be involved in regulation. It has recently been reported that MSC exosomes are most likely to function through proteins rather than miRNA [61, 62]. In conclusion, a growing number of studies have shown that EVs can promote tissue damage and repair by activating MAPK–ERK1/2 and PI3K–Akt [63–65].

This study had some limitations. First, we did not investigate increased Akt and ERK phosphorylation in RPMCs after ADSC-EV stimulation at several time points (e.g., 15, 45, and 60 min), and the time point used (30 min) might have been suboptimal. In addition, we selected tail vein injection as the method of administration. Owing to the limitation of experimental conditions, we did not verify the systemic distribution of EVS after caudal vein injection, including the distribution of the injured peritoneum. Third, although we estimated and verified the network whereby ADSC-EVs exert a functional effect through sequencing, the target miRNAs involved require further exploration. Fourth, since a large number of different miRNAs and proteins are believed to be essential for the beneficial effects of MSC-EVs, further experimental studies should identify the exact disease-specific EV source molecules responsible for the long-term protection of damaged cells. Determining the exact dose and route of EVs is our next step for future study.

## 4. Conclusions

Our work suggests that the intravenous ADSC-EV injection can significantly inhibit adhesion formation after peritoneal injury by suppressing inflammation, reducing apoptosis, and enhancing the fibrinolytic systems. Nevertheless, ADSC-EVs can significantly promote the proliferation and migration of RPMC in a dose-proportionate manner, which may be reliant on the stimulation of the MAPK–ERK 1/2 and PI3K–Akt signaling pathways (see supplementary 2). These findings support the clinical use of ADSC-EVs to manage peritoneal injuries.

## Figures and Tables

**Table 1:** Nair's macroscopic adhesion classification.

Score	Characteristics
0	Complete absence of adhesion
1	Single band of adhesion, between viscera or from viscera to abdominal wall
2	Two bands, either between the viscera or from the viscera to the abdominal wall
3	More than two bands, between the viscera or the viscera to the abdominal wall or whole intestines, form a mass without being adherent to the abdominal wall
4	Viscera directly adherent to the abdominal wall, irrespective of number and extent of adhesive bands

## Data Availability

The datasets used and/or analyzed during the current study are available from the corresponding author on reasonable request.

## Supplementary Materials

Supplementary 1 SI.1: miRNA sequencing and bioinformatics analysis of ADSC-EVs. (A) Prediction of miRNA target genes. (B) The molecular function (MF) was mainly enriched in “ATP binding.” (C) The cellular component (CC) was mainly enriched in “membrane.”

Supplementary 2 Graphical Abstract (GA). Our work suggests that the intravenous ADSC-EV injection can inhibit adhesion formation by regulating the internal environment of RPMCs in vivo. Moreover, ADSC-EVs can induce RPMC proliferation and migration by activating the MAPK–ERK1/2 and PI3K–Akt pathways.
