# Supplementary material for: Extracellular Vesicles Derived from Adipose Mesenchymal Stem Cells Promote Peritoneal Healing by Activating MAPK-ERK1/2 and PI3K-Akt to Alleviate Postoperative Abdominal Adhesion
Source: Stem Cells Int. 2022 May 5;2022:1940761. doi: 10.1155/2022/1940761 (PMC9107054; doi:10.1155/2022/1940761)
Supplement: Supplementary 1 — SI.1: miRNA sequencing and bioinformatics analysis of ADSC-EVs. (A) Prediction of miRNA target genes. (B) The molecular function (MF) was mainly enriched in “ATP binding.” (C) The cellular component (CC) was mainly enriched in “membrane.” [file 1940761.f1.docx]

**Supplementary Information:**


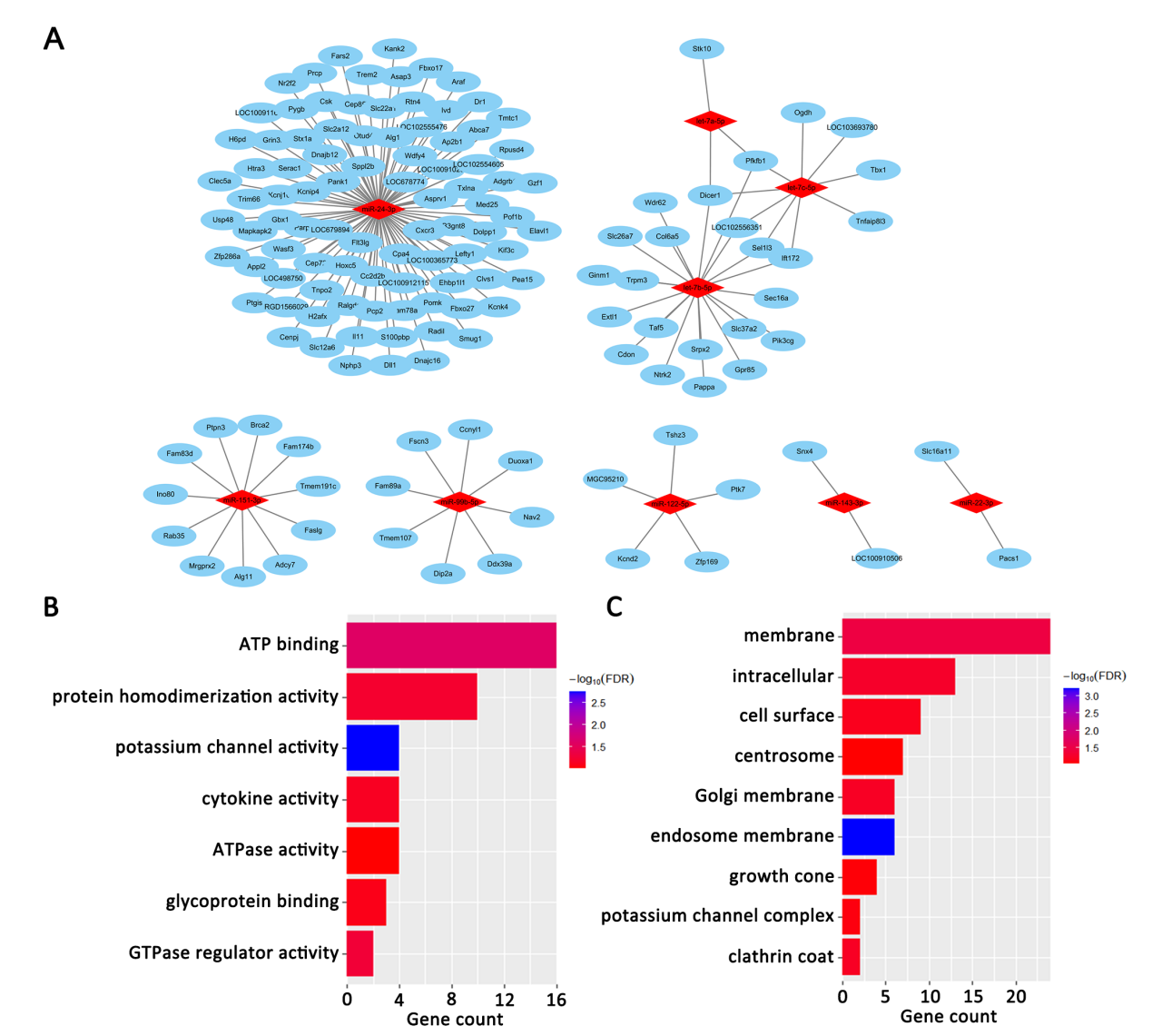


**SI.1 miRNA sequencing and bioinformatics analysis of ADSC-Exos. A**-**C** GO and KEGG pathway enrichment analyses of the possible target genes.
