# Supplementary material for: Extracellular Vesicles Derived from Adipose Mesenchymal Stem Cells Promote Peritoneal Healing by Activating MAPK-ERK1/2 and PI3K-Akt to Alleviate Postoperative Abdominal Adhesion
Source: Stem Cells Int. 2022 May 5;2022:1940761. doi: 10.1155/2022/1940761 (PMC9107054; doi:10.1155/2022/1940761)

**Graphical Abstract**

Our work suggests that the intravenous ADSC-Exo injection can inhibit adhesion formation by regulating the internal environment of RPMCs in vivo. Moreover, ADSC-Exos can induce RPMCs proliferation and migration by activating the MAPK–ERK1/2 and PI3K–Akt pathways.


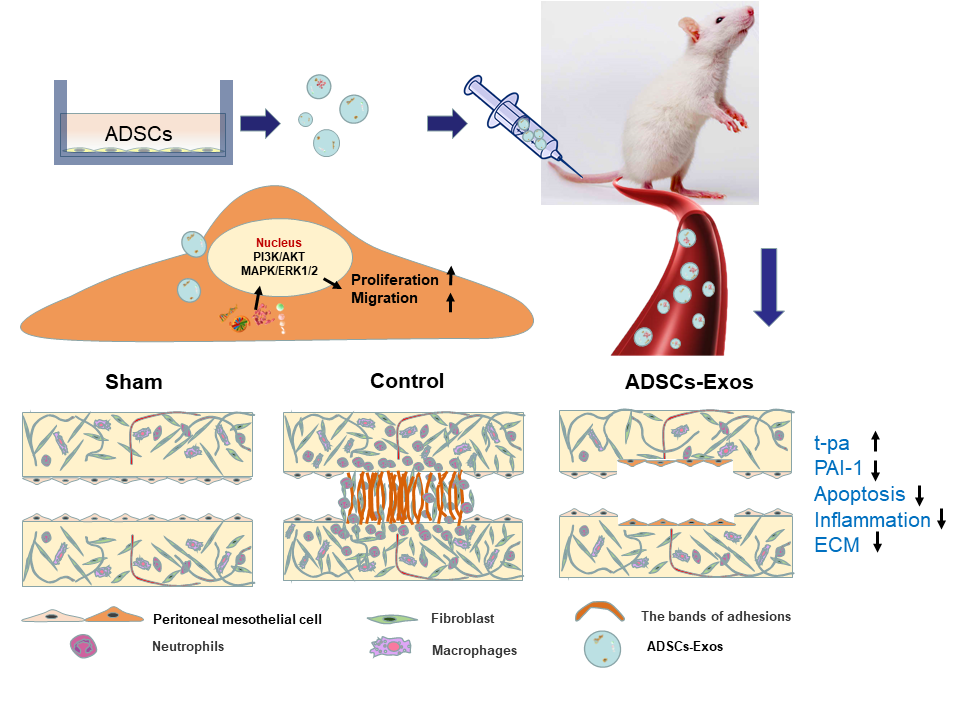

Supplement: Supplementary 2 — Graphical Abstract (GA). Our work suggests that the intravenous ADSC-EV injection can inhibit adhesion formation by regulating the internal environment of RPMCs in vivo. Moreover, ADSC-EVs can induce RPMC proliferation and migration by activating the MAPK–ERK1/2 and PI3K–Akt pathways. [file 1940761.f2.docx]
